# Supplementary material for: Dynamic Regulation of Cholesterol Metabolism Genes in Dopaminergic Neurons following Methamphetamine Treatment as Revealed by Single-Nucleus RNA Sequencing
Source: bioRxiv. 2025 Jul 31:2025.07.28.667272. Preprint. [Version 1] doi: 10.1101/2025.07.28.667272 (PMC12324295; doi:10.1101/2025.07.28.667272)
Supplement: Supplement 1 [file NIHPP2025.07.28.667272v1-supplement-1.pdf]

bioRxiv preprint doi: https://doi.org/10.1101/2025.07.28.667272; this version posted July 31, 2025. The copyright holder for this preprint (which was not certified by peer review) is the author/funder. This article is a US Government work. It is not subject to copyright under 17 USC 105 and is also made available for use under a CC0 license.

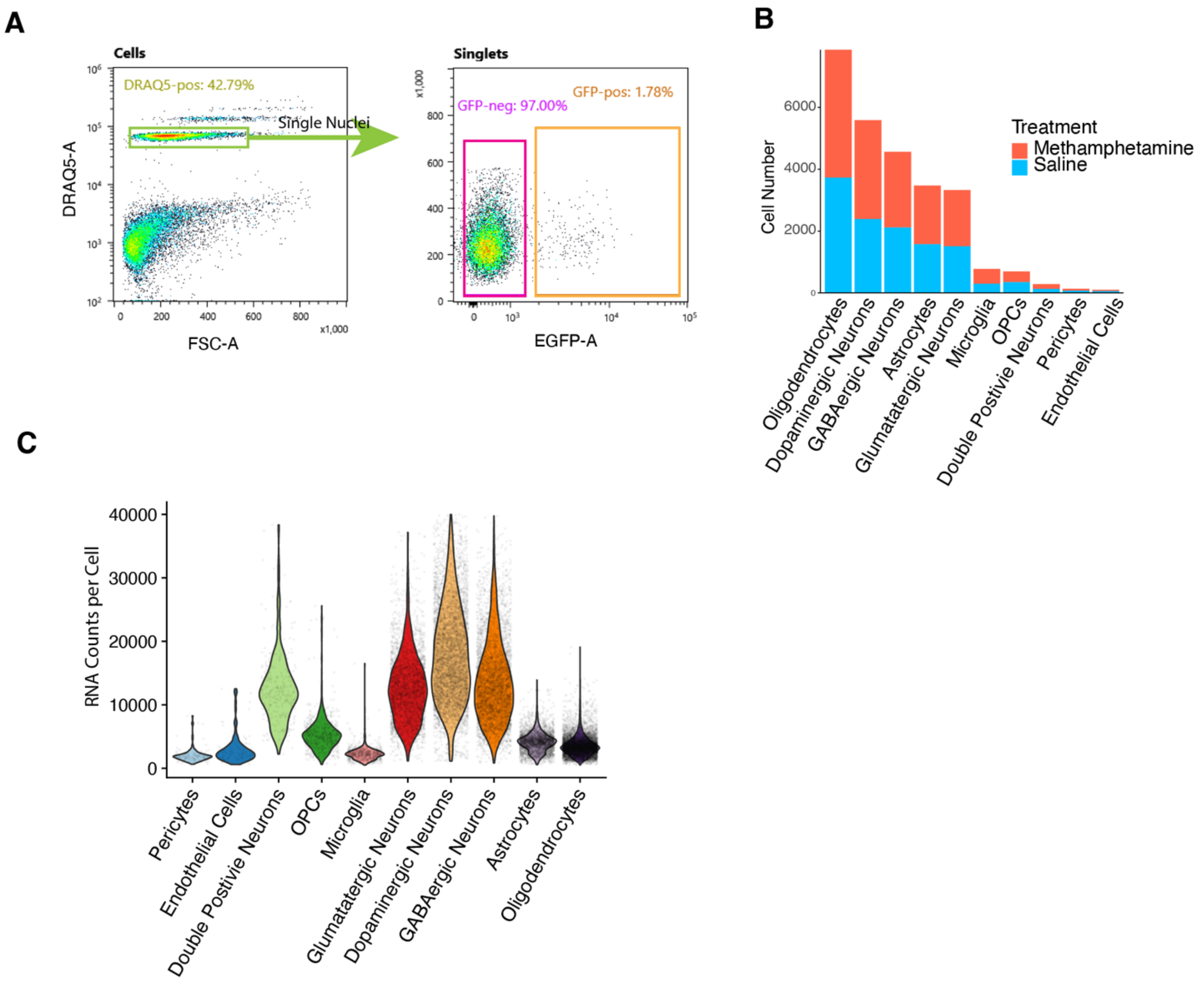

Supplementary Figure 1. Overview of single-nucleus RNA-seq data.
(A) FACS gating strategy for nuclei collection. DRAQ5^{+} single nuclei were identified and subdivided into GFP^{+} nuclei (from dopaminergic neurons) and GFP^{-} nuclei. GFP^{+} nuclei were collected first, followed by GFP^{-} nuclei, resulting in a mixed nuclei population enriched for dopaminergic neurons.
(B) Numbers of nuclei from each cell type passing quality control in the RNA-seq dataset. OPCs, oligodendrocyte precursor cells.
(C) Violin plot showing RNA counts per cell for each cell type.

bioRxiv preprint doi: https://doi.org/10.1101/2025.07.28.667272; this version posted July 31, 2025. The copyright holder for this preprint (which was not certified by peer review) is the author/funder. This article is a US Government work. It is not subject to copyright under 17 USC 105 and is also made available for use under a CC0 license.

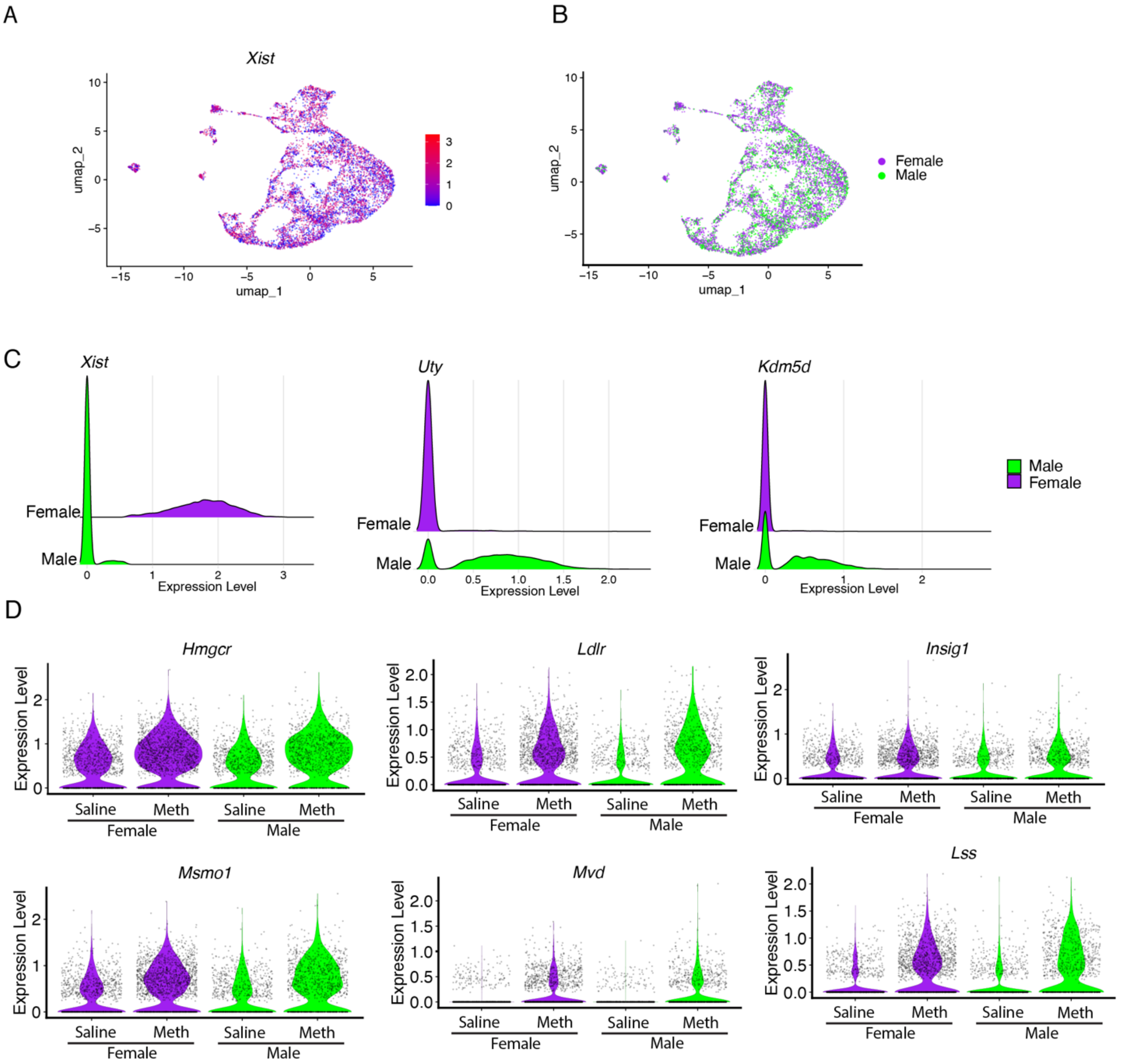

Supplementary Figure 2. Expression of cholesterol metabolism genes in male and female dopaminergic neurons.

(A) UMAP plot of dopaminergic neurons showing the distribution of *Xist* expression (female-specific marker).

(B) Separation of male and female dopaminergic neurons based on *Xist* expression.

(C) Ridge plots displaying expression of the female-specific gene *Xist* and male-specific genes *Uty* and *Kdm5d* in male and female cells.

(D) Violin plots showing expression levels of selected differentially expressed genes (DEGs) associated with cholesterol metabolism in male and female dopaminergic neurons following acute methamphetamine treatment.

bioRxiv preprint doi: https://doi.org/10.1101/2025.07.28.667272; this version posted July 31, 2025. The copyright holder for this preprint (which was not certified by peer review) is the author/funder. This article is a US Government work. It is not subject to copyright under 17 USC 105 and is also made available for use under a CC0 license.

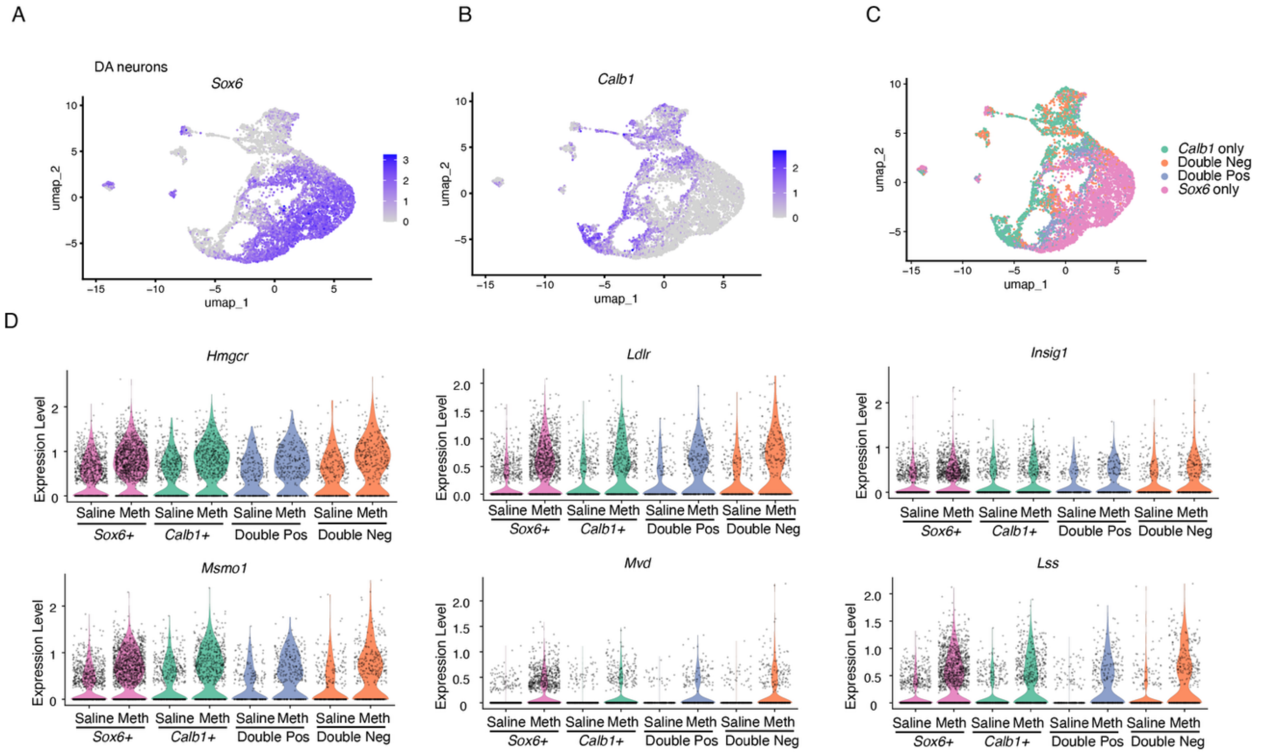

Supplementary Figure 3. Expression of cholesterol metabolism–related genes in VTA and SNc dopaminergic neuron subpopulations.

(A) UMAP plot showing expression of *Sox6*, a marker enriched in SNc dopaminergic neurons.
(B) UMAP plot showing expression of *Calb1*, a marker enriched in VTA dopaminergic neurons.
(C) UMAP plot illustrating dopaminergic neuron subpopulations divided into *Calb1*-only, *Sox6*-only, double-negative, and double-positive groups based on marker expression.
(D) Violin plots displaying expression levels of selected differentially expressed genes (DEGs) related to cholesterol metabolism in the four location-associated dopaminergic neuron subpopulations following acute methamphetamine treatment.

bioRxiv preprint doi: https://doi.org/10.1101/2025.07.28.667272; this version posted July 31, 2025. The copyright holder for this preprint (which was not certified by peer review) is the author/funder. This article is a US Government work. It is not subject to copyright under 17 USC 105 and is also made available for use under a CC0 license.

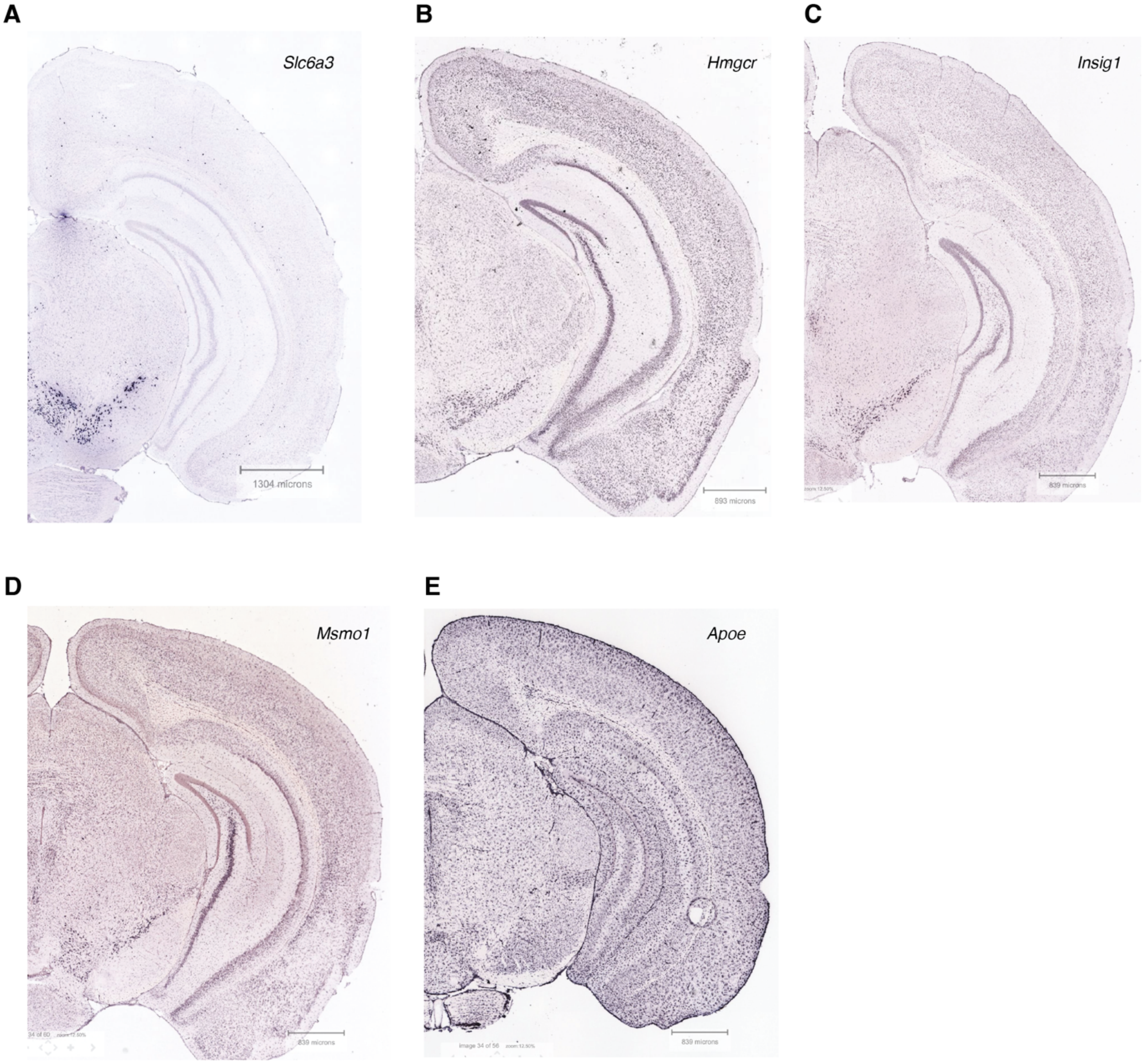

Supplementary Figure 4. *In situ* hybridization images from the Allen Brain Atlas showing distribution of genes involved in cholesterol metabolism.
(A)The expression pattern of dopaminergic marker gene *Slc6a3*.
(B-E) The expression patterns of genes involved in cholesterol metabolism.

bioRxiv preprint doi: https://doi.org/10.1101/2025.07.28.667272; this version posted July 31, 2025. The copyright holder for this preprint (which was not certified by peer review) is the author/funder. This article is a US Government work. It is not subject to copyright under 17 USC 105 and is also made available for use under a CC0 license.

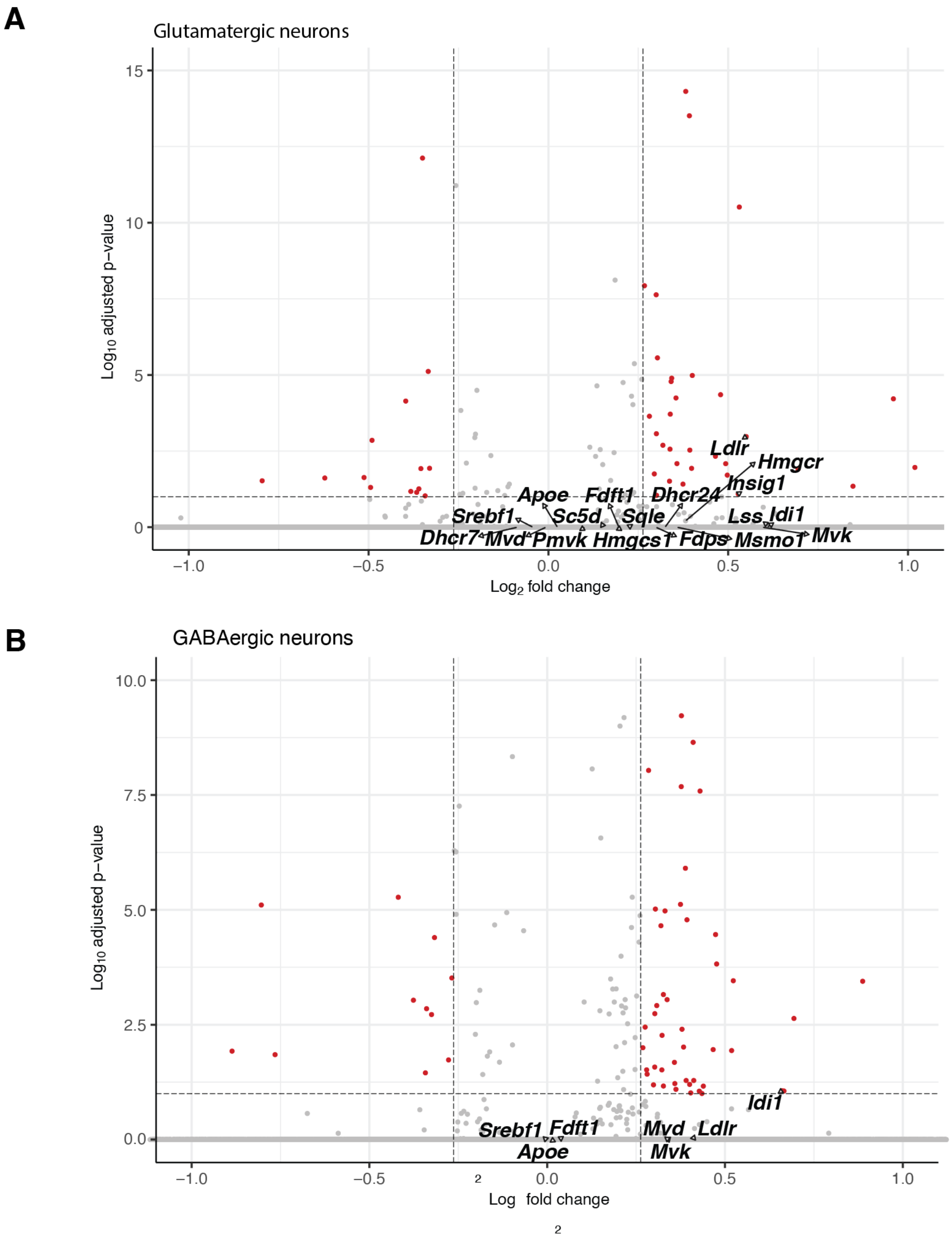

Supplementary Figure 5. Expression of cholesterol metabolism–associated genes in non-dopaminergic neurons.

(A, B) Volcano plots displaying adjusted p-values and $\log_2$ fold changes for cholesterol metabolism–related genes in glutamatergic neurons (A) and GABAergic neurons (B) following acute saline or methamphetamine treatment. Differentially expressed genes were identified using a cutoff of FDR < 0.1 and absolute fold change > 1.2.
